# Supplementary material for: Carbapenem-resistant Klebsiella pneumoniae capsular types, antibiotic resistance and virulence factors in China: a longitudinal, multi-centre study
Source: Nat Microbiol. 2024 Feb 29;9(3):814–29. doi: 10.1038/s41564-024-01612-1 (PMC10914598; doi:10.1038/s41564-024-01612-1)
Supplement: Supplementary file 1 — Supplementary Note. [file 41564_2024_1612_MOESM1_ESM.pdf]

nature microbiology

Article https://doi.org/10.1038/s41564-024-01612-1

Carbapenem-resistant *Klebsiella pneumoniae* capsular types, antibiotic resistance and virulence factors in China: a longitudinal, multi-centre study

In the format provided by the authors and unedited

Supplementary Note

Antimicrobial profiling of CRKP isolates during 2016-2020

Antimicrobial susceptibility testing was performed to determine the sensitive of the CRKP isolates to different antibiotics, and the results showed that 99.4% and 98.9% of 1017 CRKP isolates were resistant to the carbapenems antibiotics meropenem and imipenem, respectively. The susceptibility rates of polymyxin B, tigecycline, and ceftazidime-avibactam were 94.9%, 94.4%, and 92.1%, respectively (Supplementary Table 2), which showed potent *in vitro* activity against CRKP isolates. The trimethoprim-sulfamethoxazole and amikacin showed limited *in vitro* activity against CRKP isolates with 36.4% susceptibility and 30.5% susceptibility, respectively, and the susceptibility rates of other agents were <3%, including cephalosporins and fluoroquinolones agents (Supplementary Table 2). Genomic analysis revealed that 44.0% (11/25) of polymyxin B-resistant isolates had mutations in the *mgrB* gene among the KL64 and KL47 strains, and these *mgrB*-mutated CRKP isolates were ST11 (Supplementary Table 5). Four isolates showed resistant to tigecycline carrying a specific *tet*(A)-variant (Supplementary Table 5). The *mgrB* mutation in polymyxin B-resistant CRKP isolates were associated with its reduced susceptibility to polymyxin B. Also, mutations in *tetA* genes may result in lower tigecycline susceptibility. However, further research is necessary to validate this hypothesis.

Virulence plasmids of KL64 and KL47 strains

Over 90% (550/595) of hv-CRKPs isolates in KL64 were associated with a pLVPK-similar plasmid (subsequently named pST11-Vir-1-KPC), which carries 2-5 hypervirulence-associated genes. It was acquired by KL64 in 2006 (CI95% 2005-2007) and subsequently maintained stably (Figure 3B). In contrast, apart from a few KL47 isolates from Zhejiang, other hv-CRKPs in KL47 were attributed to two other plasmids of pST11-Vir-2 and pST11-Vir-3, which carried only *iucA* and *rmpA2*. Furthermore, no major hv-CRKPs cluster was identified in KL47, and the virulence plasmids were only regionally maintained (Figure 3B).

The variations of hv-CRKP in KL64 and KL47 strains were associated to certain virulence plasmids in this study. Most of the KL64 hv-CRKPs were attributed to a single acquisition of the pST11-Vir-1-KPC plasmid (Fig. 3B), which encodes 2-5 hypervirulence genes and is frequently

fused with carbapenemase-carrying plasmids[1]. However, the majority of KL47 hv-CRKPs were associated with two other plasmids (pST11-Vir-2 and pST11-Vir-3) that carried only *iucA* and *rmpA2*. These hv-CRKP cluster with certain virulence plasmids had attributed to the increase rate of KL47 between Beijing, Henan and Liaoning in the northern China, while the resting strains without virulence plasmids were mainly transmitted into Sichuan and Hubei in the southern China. Such different evolutionary dynamics of hv-CRKP strains after the acquisition of virulence plasmids revealed a complex interplay between virulence plasmids, the core genome, and the geography in the emergence and spread of hypervirulent carbapenem-resistance.

1 Yang, X., Dong, N., Chan, E. W., Zhang, R. & Chen, S. Carbapenem Resistance-Encoding and Virulence-Encoding Conjugative Plasmids in Klebsiella pneumoniae. *Trends Microbiol* **29**, 65-83, doi:10.1016/j.tim.2020.04.012 (2021).
