# Supplementary material for: Carbapenem-resistant Klebsiella pneumoniae capsular types, antibiotic resistance and virulence factors in China: a longitudinal, multi-centre study
Source: Nat Microbiol. 2024 Feb 29;9(3):814–29. doi: 10.1038/s41564-024-01612-1 (PMC10914598; doi:10.1038/s41564-024-01612-1)
Supplement: Supplementary file 2 — Reporting Summary [file 41564_2024_1612_MOESM2_ESM.pdf]

nature portfolio

Corresponding author(s): He Ping, Zhemin Zhou

Last updated by author(s): Jan 12, 2024

Reporting Summary

Nature Portfolio wishes to improve the reproducibility of the work that we publish. This form provides structure for consistency and transparency in reporting. For further information on Nature Portfolio policies, see our Editorial Policies and the Editorial Policy Checklist.

Statistics

For all statistical analyses, confirm that the following items are present in the figure legend, table legend, main text, or Methods section.

n/a	Confirmed	
☐	☒	The exact sample size (*n*) for each experimental group/condition, given as a discrete number and unit of measurement
☐	☒	A statement on whether measurements were taken from distinct samples or whether the same sample was measured repeatedly
☐	☒	The statistical test(s) used AND whether they are one- or two-sided<br>*Only common tests should be described solely by name; describe more complex techniques in the Methods section.*
☒	☐	A description of all covariates tested
☒	☐	A description of any assumptions or corrections, such as tests of normality and adjustment for multiple comparisons
☐	☒	A full description of the statistical parameters including central tendency (e.g. means) or other basic estimates (e.g. regression coefficient) AND variation (e.g. standard deviation) or associated estimates of uncertainty (e.g. confidence intervals)
☐	☒	For null hypothesis testing, the test statistic (e.g. *F*, *t*, *r*) with confidence intervals, effect sizes, degrees of freedom and *P* value noted<br>*Give P values as exact values whenever suitable.*
☒	☐	For Bayesian analysis, information on the choice of priors and Markov chain Monte Carlo settings
☒	☐	For hierarchical and complex designs, identification of the appropriate level for tests and full reporting of outcomes
☒	☐	Estimates of effect sizes (e.g. Cohen's *d*, Pearson's *r*), indicating how they were calculated

Our web collection on statistics for biologists contains articles on many of the points above.

Software and code

Policy information about availability of computer code

Data collection	Using SRA Toolkit (2.8.0) to download genomes and short reads from NCBI
Data analysis	The following software were used in the analysis: Trimmomatic v0.39, EToKi v1.3, PROKKA v1.14, ISfinder v1, Kleborate v2.3.2, Kaptive v2.0.7, RecHMM v1, Phandango v1.3.0, BactDating v1.1, TreeTime v0.10.1, iTOL v6, TempEst v1.5.3, BLASTn v2.9.0+, MOB-Typer v3.1.0, BRIG v0.95, BBDuk v1, KRAKEN v2.1.2, minimap2 v2.26-r1175, samtools v1.2, SPARSE v1, R skygrowth v0.3.1, R APE v5.7.1, LINcoding script v1, and search plasmic.git v1 (https:/eithub.com/xiaoliu8 /search plasmic.git)

For manuscripts utilizing custom algorithms or software that are central to the research but not yet described in published literature, software must be made available to editors and reviewers. We strongly encourage code deposition in a community repository (e.g. GitHub). See the Nature Portfolio guidelines for submitting code & software for further information.

Data

Policy information about availability of data

All manuscripts must include a data availability statement. This statement should provide the following information, where applicable:

- Accession codes, unique identifiers, or web links for publicly available datasets
- A description of any restrictions on data availability
- For clinical datasets or third party data, please ensure that the statement adheres to our policy

The raw reads of 542 K. pneumoniae sequenced in this study were uploaded to NCBI database under the project PRJNA1028672. A total of 3,047 publicly available

genomes were enrolled including 2,695 public ST11 K. pneumoniae genomes which were available in NCBI database under various projects (see supplementary table 6), and 352 ST11 K. pneumoniae genomes were downloaded from Chinese Genome Sequence Archive in National Genomics Data Center under project PRJCA003173 (n=300) and PRJCA012323 (n=52). The metagenomic sequences of Clinical samples acquired during the capsule and O-Antigen trial parts were uploaded to NCBI database under the project PRJNA1028672. The transcriptome sequencing data were uploaded to NCBI database under the project PRJNA1028672 as well. Reference genome for phylogenetic analysis of ST11 strains were set as GCF_011066505.1

Research involving human participants, their data, or biological material

Policy information about studies with human participants or human data. See also policy information about sex, gender (identity/presentation), and sexual orientation and race, ethnicity and racism.

Reporting on sex and gender	Sex and gender were not considered in study design.
Reporting on race, ethnicity, or other socially relevant groupings	Socially constructed or socially relevant categorization variable(s) were not used in our manuscript.
Population characteristics	A total of 1,017 CRKP strains were collected from different specimen sources (sputum, urine, blood, etc.) of K. pneumoniae infected patients admitted to 40 hospitals in 26 different Chinese cities between 2016-2020. More than half of the patients were male (667/1017; 65.59%). The mean age of the patients was 56.17 (SD 18.70). Additional information on patients was not available to the authors. The sputum, urine, bile and pus used for metagenomic sequencing were collected from K. pneumoniae infected patients, 83.3% (10/12) of whom were male, and the mean age of whom was 66.00 (SD 11.59). The serum of K. pneumoniae infected patients or healthy volunteers for SBA assay were also collected at Ruijin Hospital affiliated with Shanghai Jiaotong University but no information about patients or healthy volunteers was available.
Recruitment	Patients were recruited from the 40 member hospitals of CHINET China Bacterial Drug Resistance Surveillance Network (CHINET) every October 1-31 between 2016-2020. During this period, consecutive non-repetitive isolates were collected from all patients infected with K. pneumoniae. For multiple K. pneumoniae isolates from the same patient, the initial isolate was selected. Antimicrobial susceptibility testing was then performed to identify CRKP strains, and finally CRKP-infected patients were recruited. For the metagenomic sequencing and SBA assay, recruitment patients were approached at the time of diagnosis of K. pneumoniae infection and asked if they would like to participant in this study. Willing patients were recruited and samples were collected from each patient. There was no selection of infected patients, so there is no potential bias that could affect the outcome of the study.
Ethics oversight	The study protocol was approved by the Institutional Review Board of Huashan Hospital, Fudan University (No. 2018-408 and No.2019-460). Patients' serum, sputum, urine, bile and pus were collected at Ruijin Hospital affiliated with Shanghai Jiaotong University and approval was obtained from the Ethics Committee of the Ruijin Hospital, Shanghai Jiaotong University (No.2017-205).

Note that full information on the approval of the study protocol must also be provided in the manuscript.

Field-specific reporting

Please select the one below that is the best fit for your research. If you are not sure, read the appropriate sections before making your selection.

☒ Life sciences ☐ Behavioural & social sciences ☐ Ecological, evolutionary & environmental sciences

For a reference copy of the document with all sections, see nature.com/documents/nr-reporting-summary-flat.pdf

Life sciences study design

All studies must disclose on these points even when the disclosure is negative.

Sample size	We collected a total of 1,017 CRKP strains between 2016 and 2020 in China. Then we selected 526 strains for whole genome sequencing based on sequencing and selecting criteria (see Extended Data Fig.1). In addition, we downloaded a total of 3,047 K. pneumoniae strains (Sequence type 11) from public databases, including 2,695 assembled genomes from NCBI SRA database, and 352 assembled genomes from Genome Sequence Archive in National Genomics Data Center, China.<br><br>We did PCR verification on 1,017 CRKPs in the carbapenem resistance experiment and discovered 18 non-carbapenemase-producing CRKP strains. Of which, 2 bacteria have been included in the 526 sequenced strains, thus we performed Whole Genome Sequencing on the other 16 bacteria.<br><br>Clinical samples were acquired during the capsule and O-Antigen trial parts. Only 12 clinical samples passed the sequencing standards after DNA extraction and quality control, thus metagenomic sequencing was done on these 12 samples.<br><br>We conducted capsule Quellung tests on KL64 and KL47 strains in 1,017 CRKPs and discovered that 70 strains were non-capsulated (NEKp group); consequently, based on the selection criteria (see Extended Data Fig.1), 70 encapsulated strains (EKp group) were chosen as controls for fitness testing.
Data exclusions	No data were excluded from the analyses.
Replication	Numbers of experimental replications were stated in the figure legend. All attempts at replication were successful. Bioinformatic analyses

Replication	were described in sufficient detail to reproduce the findings with the publicly available sequence data. We have given out all the source data as described in the manuscript. And the required scripts have been released in github, which has been described in the manuscript.
Randomization	For the mice experiment, mice with same age and gender used for evaluating the systemic infectivity of the bacteria were randomly allocated into different groups.<br>For the strategy of whole genome sequencing, we have set up a sequencing criterion (see Extended Data Fig.1), and we sequenced all strains that met the criteria.<br>For the fitness experiment, we conducted capsule Quellung tests on all KL64 and KL47 strains and discovered that 70 strains were non-capsular. Then, we selected 70 encapsulated strains randomly under the selection criteria of similar isolation regions and sources as showed in Extended Data Fig.1.
Blinding	For the mice experiment, investigators were blinded for evaluating the systemic infectivity of the bacteria, but no blinding was performed for the bacterial competition assay in mice because of the experimental complexity and group comparisons were not involved in this experiment. In other experiments, investigators were blinded to group allocation during data collection and analysis. In the part of bioinformatic analysis, blinding was unnecessary since this study is not a clinical trial.

Reporting for specific materials, systems and methods

We require information from authors about some types of materials, experimental systems and methods used in many studies. Here, indicate whether each material, system or method listed is relevant to your study. If you are not sure if a list item applies to your research, read the appropriate section before selecting a response.

Materials & experimental systems

n/a	Involved in the study	
☐	☒	Antibodies
☐	☒	Eukaryotic cell lines
☒	☐	Palaeontology and archaeology
☐	☒	Animals and other organisms
☒	☐	Clinical data
☒	☐	Dual use research of concern
☒	☐	Plants

Methods

n/a	Involved in the study	
☒	☐	ChIP-seq
☒	☐	Flow cytometry
☒	☐	MRI-based neuroimaging

Antibodies

Antibodies used	K47-antiserum and K64-antiserum (antibodies) used for Quellung Reaction were prepared by ourselves due to the limited availability of commercial antibodies.
Validation	K47-antiserum and K64-antiserum were confirmed against the capsule of K47 and K64 strains, respectively, in our previous studies (https://doi.org/10.1128/msphere.00271-22, https://doi.org/10.3390/antibiotics10020144).

Eukaryotic cell lines

Policy information about cell lines and Sex and Gender in Research

Cell line source(s)	Murine macrophage cell line RAW 264.7 (ATCC Cat#TIB-71) was used in the study.
Authentication	The cell line was not authenticated.
Mycoplasma contamination	The cell line was not tested for mycoplasma contamination.
Commonly misidentified lines (See ICLAC register)	No commonly misidentified cell lines were used in the study.

Animals and other research organisms

Policy information about studies involving animals; ARRIVE guidelines recommended for reporting animal research, and Sex and Gender in Research

Laboratory animals	All animal experiments were performed in female 7-week-old C57BL/6 mice.
Wild animals	This study did not involve wild animals.
Reporting on sex	Sex was not considered in study design.
Field-collected samples	This study did not involve samples collected from the field.

Ethics oversight

All animal procedures were performed in accordance with the Regulations for the Administration of Affairs Concerning Experimental Animals and approved by the Animal Ethics Review Committee of School of Pharmacy, Fudan University (Project Number 2023-03-HSYY-QXH-31) and the Animal Ethics Review Committee of Shanghai Jiaotong University (project number A-2023-009).

Note that full information on the approval of the study protocol must also be provided in the manuscript.

Plants

Seed stocks

Report on the source of all seed stocks or other plant material used. If applicable, state the seed stock centre and catalogue number. If plant specimens were collected from the field, describe the collection location, date and sampling procedures.

Novel plant genotypes

Describe the methods by which all novel plant genotypes were produced. This includes those generated by transgenic approaches, gene editing, chemical/radiation-based mutagenesis and hybridization. For transgenic lines, describe the transformation method, the number of independent lines analyzed and the generation upon which experiments were performed. For gene-edited lines, describe the editor used, the endogenous sequence targeted for editing, the targeting guide RNA sequence (if applicable) and how the editor was applied.

Authentication

Describe any authentication procedures for each seed stock used or novel genotype generated. Describe any experiments used to assess the effect of a mutation and, where applicable, how potential secondary effects (e.g. second site T-DNA insertions, mosiacism, off-target gene editing) were examined.
