# Supplementary figures and images for: Carbapenem-resistant Klebsiella pneumoniae capsular types, antibiotic resistance and virulence factors in China: a longitudinal, multi-centre study
Source: Nat Microbiol. 2024 Feb 29;9(3):814–29. doi: 10.1038/s41564-024-01612-1 (PMC10914598; doi:10.1038/s41564-024-01612-1)

Figure 6B

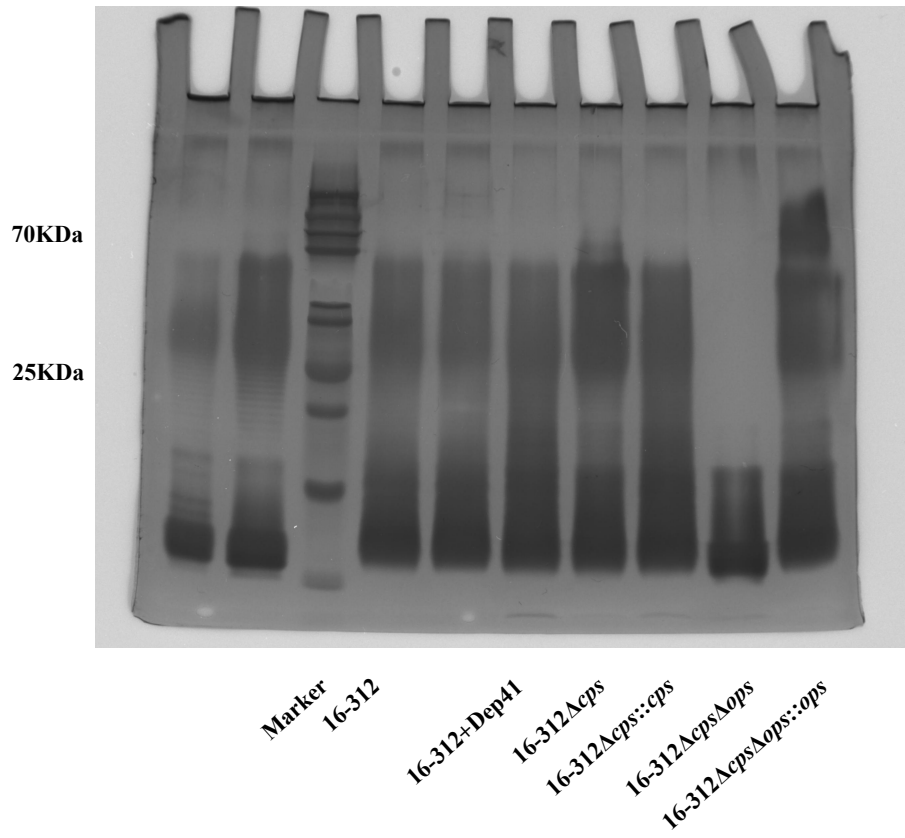

Supplement: Supplementary file 8 — Unprocessed gels for Fig. 6. [file 41564_2024_1612_MOESM8_ESM.pdf]

Extended Figure 7

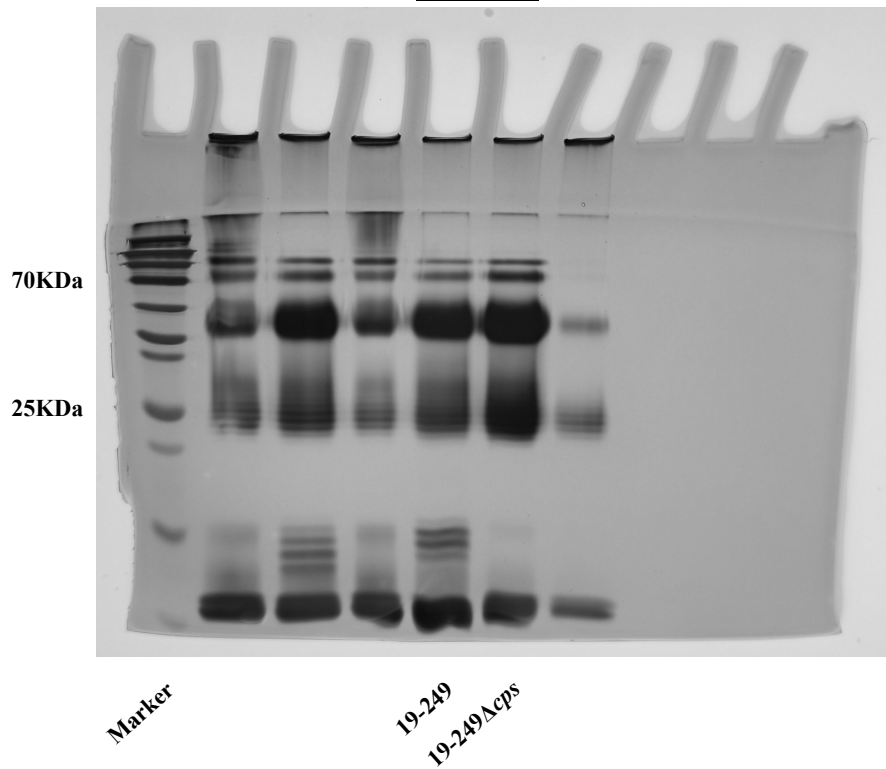

Extended Figure 7

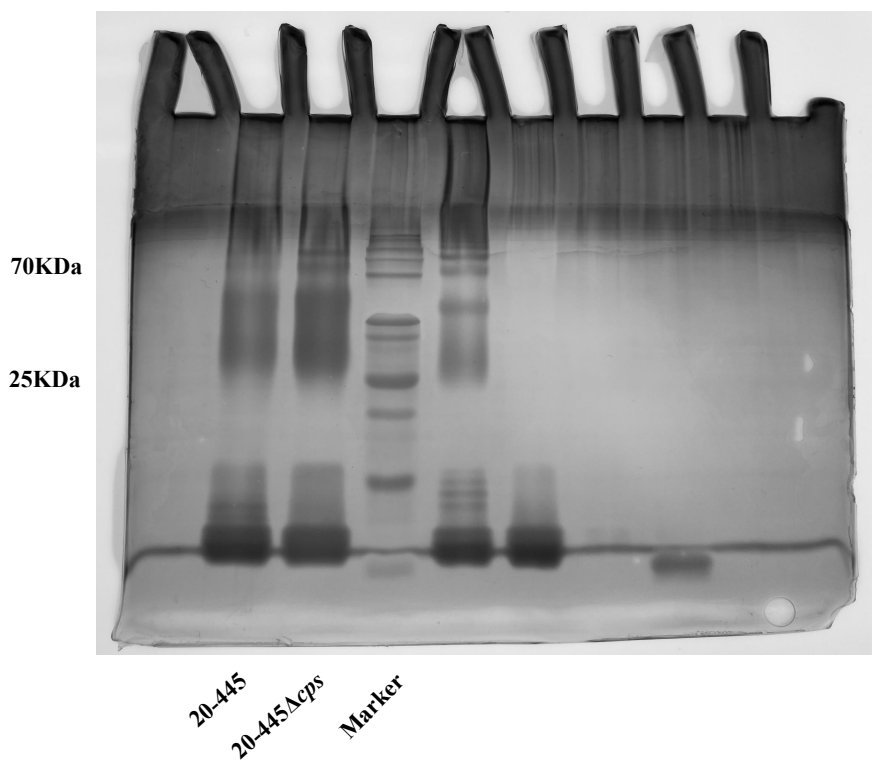

Extended Figure 7

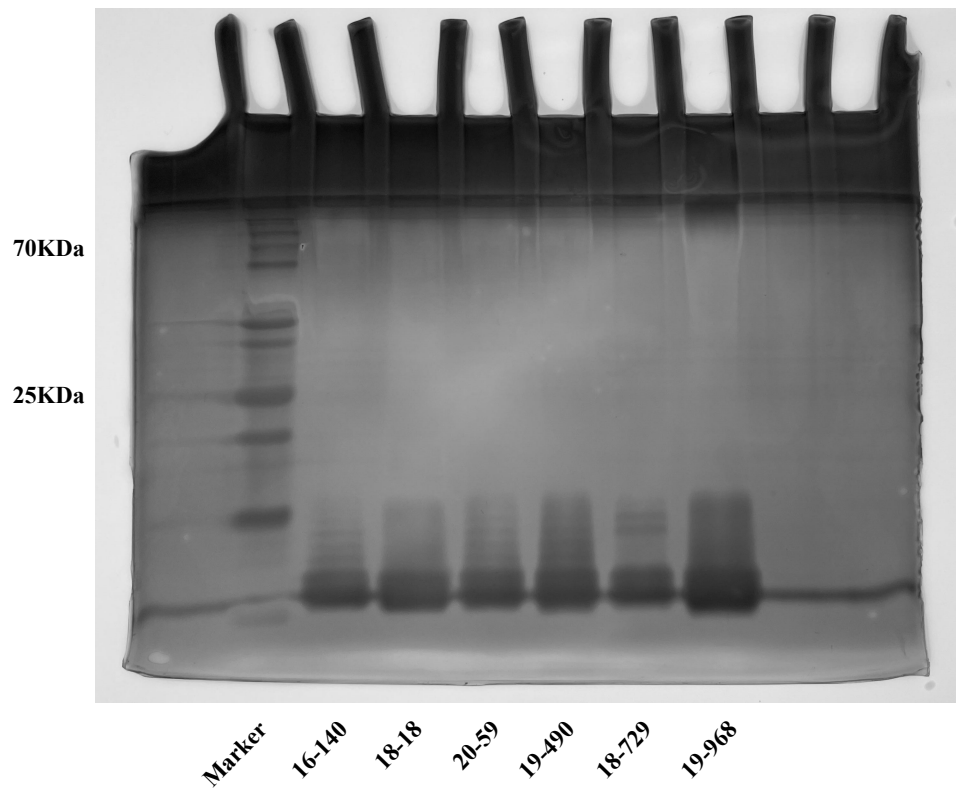

Supplement: Supplementary file 12 — Unprocessed gels for Extended Data Fig. 7. [file 41564_2024_1612_MOESM12_ESM.pdf]
