# Supplementary material for: Carbapenem-resistant Klebsiella pneumoniae capsular types, antibiotic resistance and virulence factors in China: a longitudinal, multi-centre study
Source: Nat Microbiol. 2024 Feb 29;9(3):814–29. doi: 10.1038/s41564-024-01612-1 (PMC10914598; doi:10.1038/s41564-024-01612-1)

Extended Figure 9C

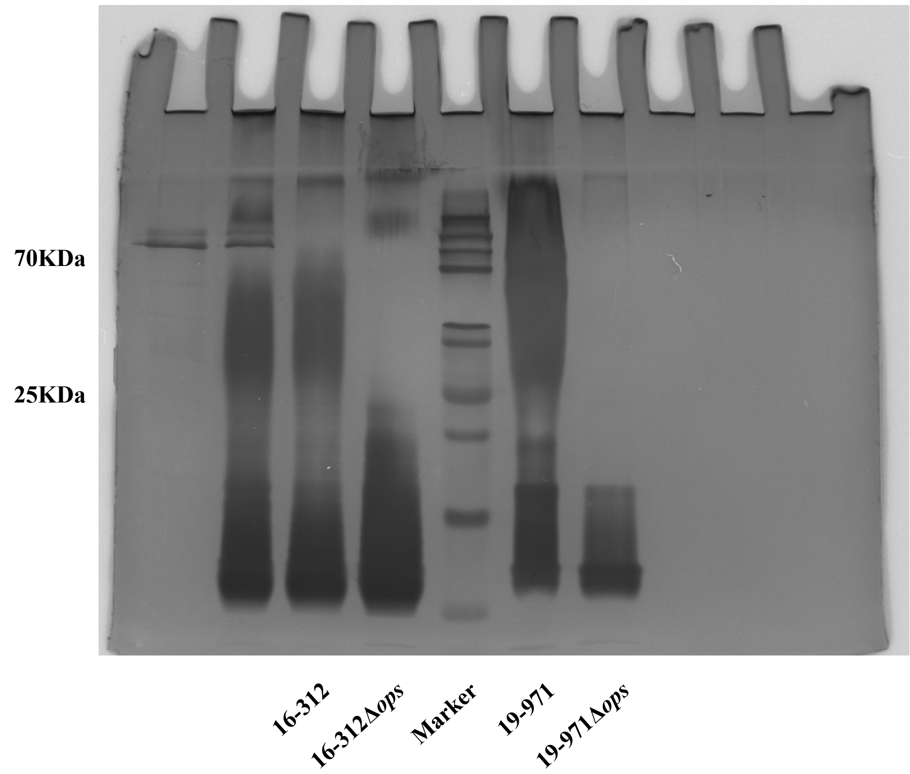

Extended Figure 9C

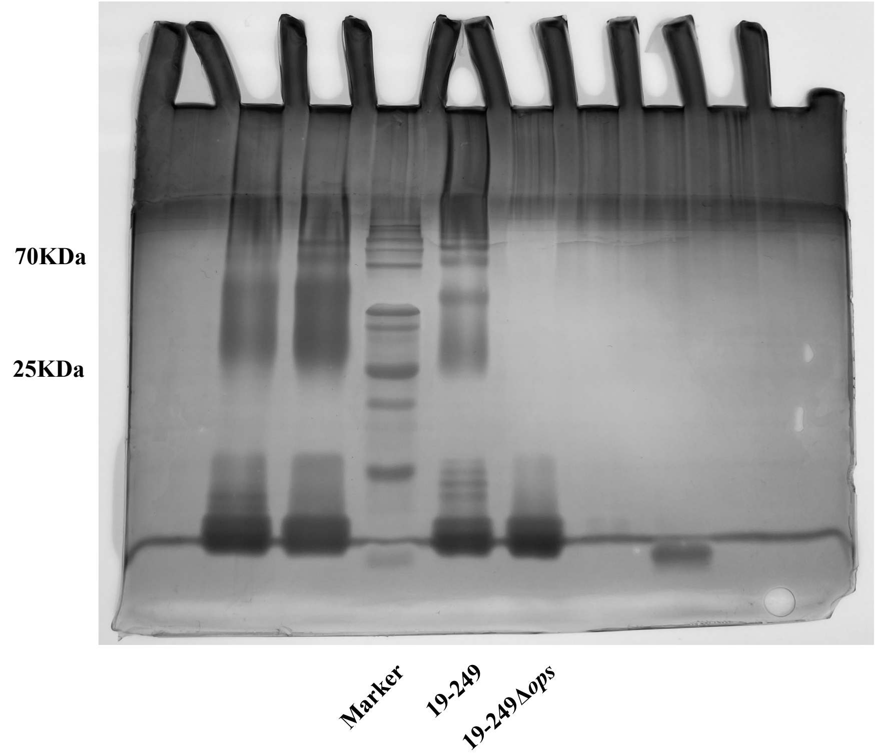

Extended Figure 9C

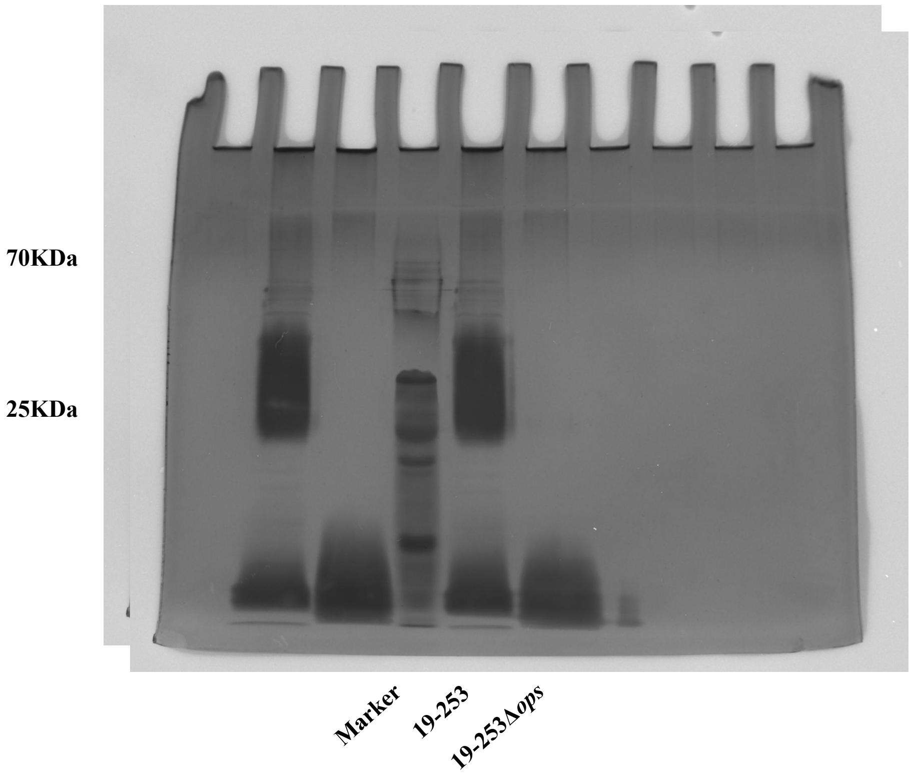

Extended Figure 9D

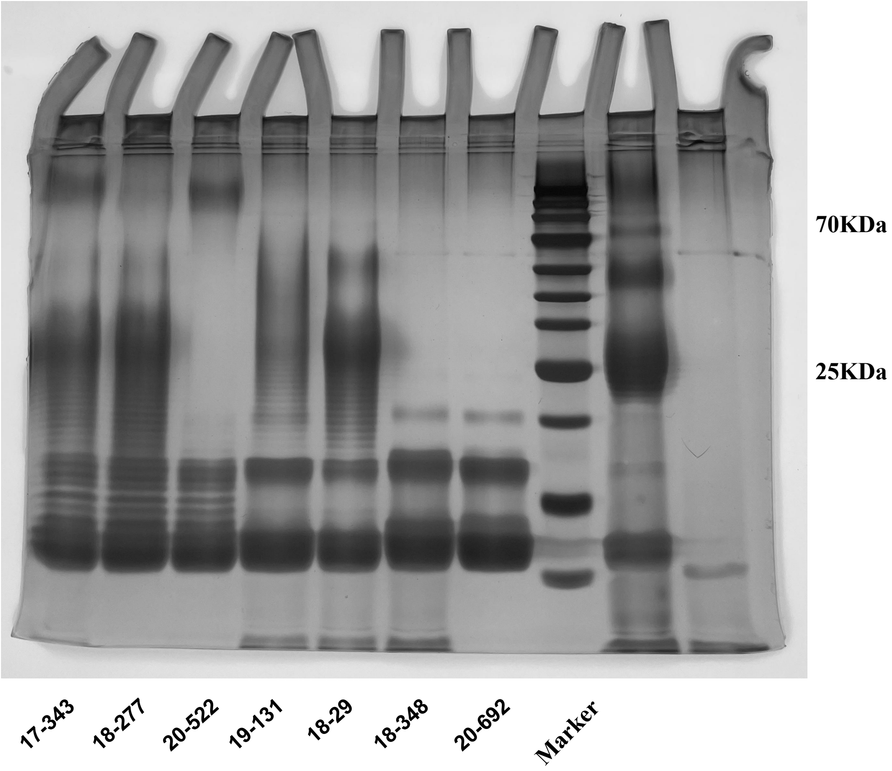

Extended Figure 9D

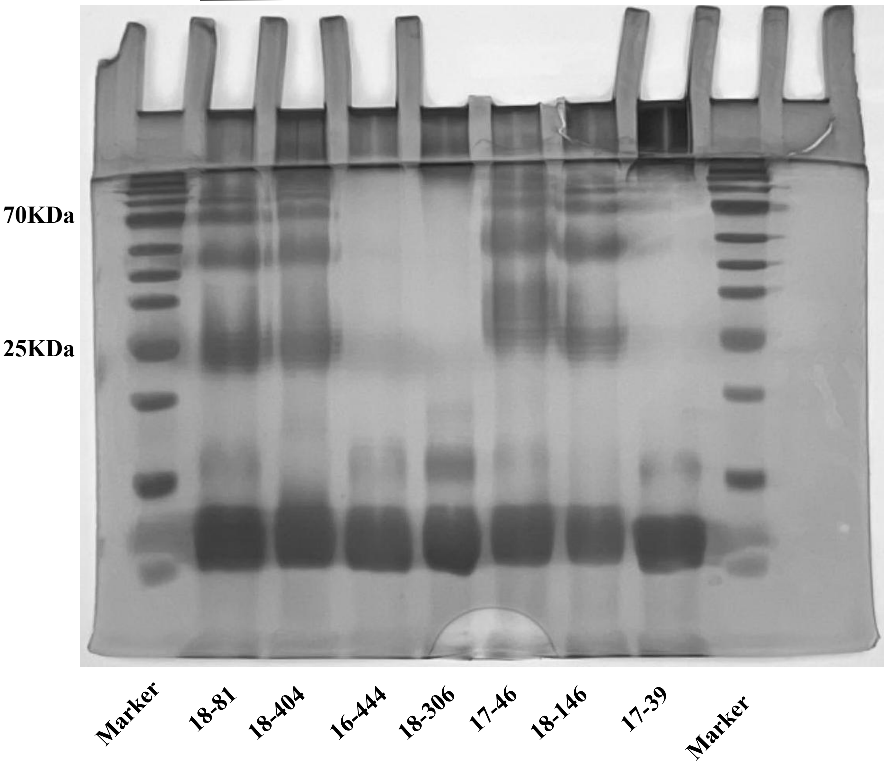

Extended Figure 9E

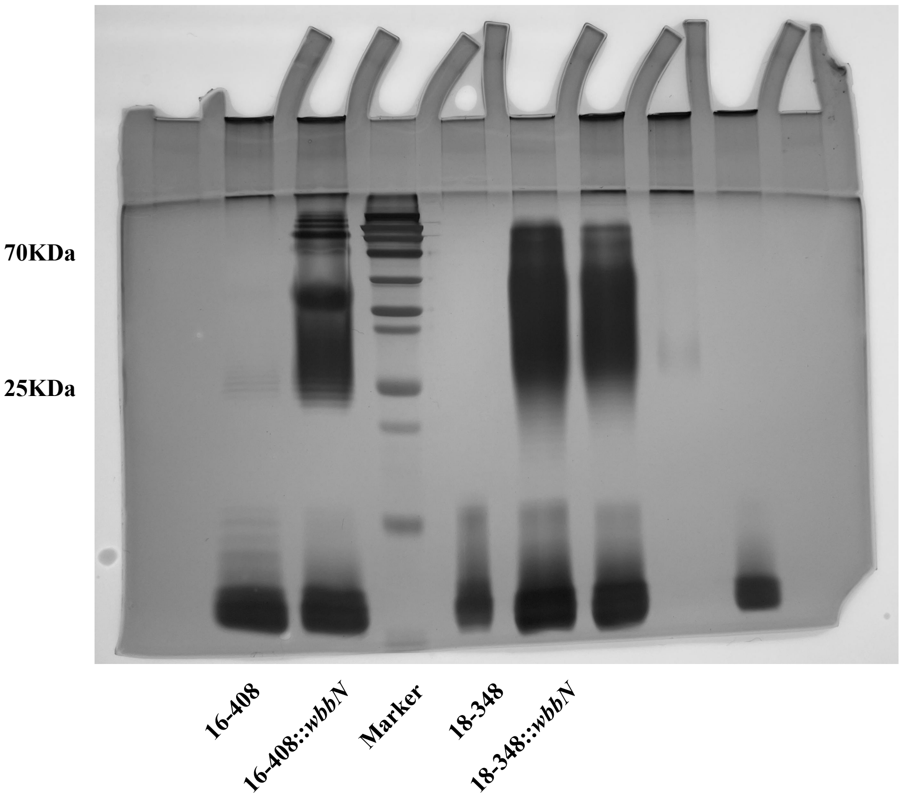

Supplement: Supplementary file 14 — Unprocessed gels for Extended Data Fig. 9. [file 41564_2024_1612_MOESM14_ESM.pdf]
